# Prevalence of HDV, HCV, and HIV Infection in the Population of Patients Infected with HBV in a Romanian Cohort

**DOI:** 10.3390/microorganisms13010118

**Published:** 2025-01-09

**Authors:** Antoanela Curici, Olivia Mioara Ilie, Dana Elena Mindru

**Affiliations:** 1 Department of Cellular and Molecular Biology and Histology, “Carol Davila” University of Medicine and Pharmacy, 050474 Bucharest, Romania; 2 Synevo Romania, 021408 Bucharest, Romania; 3 Department of Pediatrics, Faculty of Medicine, “Grigore T. Popa” University of Medicine and Pharmacy, 700115 Iasi, Romania; mindru.dana@umfiasi.ro

**Keywords:** hepatitis B, hepatitis delta, prevalence, liver enzyme, HCV, HIV

## Abstract

Hepatitis B virus (HBV) infections remain a significant global health challenge, especially in low- and middle-income countries where access to healthcare services is often limited. This study aimed to assess the prevalence of hepatitis B virus (HBV), hepatitis delta virus (HDV), hepatitis C virus (HCV), and human immunodeficiency virus (HIV) co-infections in a cohort of 426,528 patients tested for HBsAg in Romania between 2018 and 2023. Of the 17,082 HBsAg-positive individuals (4.0% prevalence), the highest HBV positivity rates were observed in the 30–39 and over 60 age groups. Chronic HBV infection was identified in 13.2% of the cohort, with 3.6% testing positive for HBeAg, indicating active viral replication. Co-infection rates were 11.3% for HDV, 1.4% for HCV, and 0.45% for HIV. The incidence of HDV co-infection increased significantly from 2018 to 2023, particularly in older populations. HCV co-infection was more prevalent in individuals aged 50–59 and over 60, with a declining trend from 2020 onward. The study also revealed a weak correlation between liver enzyme levels (ALT and AST) and HBV viral load, suggesting that liver function tests may not fully reflect the severity of HBV infection. HIV co-infection was notably rare compared to other regions, likely due to regional healthcare interventions. The findings from our study highlight the need for targeted interventions, particularly for high-risk groups such as older adults and middle-aged individuals, to reduce the burden of chronic HBV and its complications.

## 1. Introduction

The latest report of the World Health Organization (WHO) on viral hepatitis epidemiology, covering data from 187 countries across the world, shows that despite global efforts, viral hepatitis remains a major public health challenge of this decade, and it is still off track toward 2030 Sustainable Development Goals [1]. Although the estimated number of people newly infected by viral hepatitis declined from 3 million in 2019 to 2.2 million in 2022, with 1.2 million of these caused by new infections with hepatitis B, the burden of viral hepatitis is still high, being one of the communicable diseases for which the number of hepatitis-related cancer cases and deaths are increasing. At the end of 2022, 254 million people were living with chronic hepatitis B worldwide. The mortality global rate for all viral infections increased from 1.1 million deaths in 2019 to 1.3 million in 2022, out of which hepatitis B alone was responsible for 83% of the total death cases. Despite the availability of an effective vaccine and antiviral therapies, HBV continues to contribute substantially to the global burden of this disease, particularly in low- and middle-income countries where access to healthcare services is limited [1].

When addressing the pediatric population concerns, according to the World Health Organization (WHO), the majority of B chronic hepatitis (HBV) infections are acquired perinatally or during early childhood. The organization advocates for the timely administration of the birth dose (TBD) vaccine to newborns within the first 24 h of life. Following the introduction of the vaccine, the WHO has observed a decline in HBV prevalence among children under 5 years of age, reducing it from 5% to less than 1% [2].

The epidemiology of HBV infection exhibits significant geographical variability. The highest prevalence rates are observed in the Western Pacific and African regions, where prevalence among the general population exceeds 5%. In contrast, the prevalence is lower in the Americas and Europe, where rates are generally below 2%. The disease burden for hepatitis B remains high in the EU/EEA. The number of newly diagnosed hepatitis B cases is also high, even though the EU/EEA is a low-endemicity area. Most reported cases in 2022 were chronic hepatitis B infections [3].

Most HBV infections worldwide are acquired through perinatal transmission at birth, horizontal transmission among young children, sexual contact, and injecting drugs. Although control measures have significantly reduced transmission through contaminated blood and unsafe medical practices, healthcare-associated infections remain a major concern in both resource-limited and well-resourced environments [4].

The clinical management is further complicated by co-infections with other hepatotropic viruses and HIV [5]. The co-infection of hepatitis D virus (HDV) and hepatitis B virus (HBV) has been shown to significantly worsen the progression of chronic viral hepatitis, displaying features of chronic hepatitis such as necroinflammation and fibrosis [1]. This condition leads to increased liver damage and a heightened risk of hepatocellular carcinoma, resulting in a faster decline in health and, ultimately, a greater likelihood of death.

Co-infection with hepatitis B virus (HBV) and hepatitis C virus (HCV) with an estimated global prevalence ranging from 1% to 15% results in faster disease progression and a higher incidence of hepatocellular carcinoma compared to single infections [6,7]. Similarly, HIV/HBV co-infection, due to shared transmission routes, leads to more rapid liver disease progression, elevated risks of liver cancer, liver-related complications, and increased mortality. Consequently, screening for HBV and timely treatment are essential for individuals living with HIV [8].

This study aimed to characterize the epidemiology of hepatitis B virus (HBV) infections in Romania, analyzing data to assess prevalence, age and gender distribution, and co-infection rates with hepatitis C, hepatitis D, and HIV over a five-year period. By examining the HBsAg-positive population in Romania from 2018 to 2023, this study sought to illuminate trends in HBV infection rates, demographic variations, and the impact of co-infections, providing critical insights for targeted public health interventions.

## 2. Materials and Methods

### 2.1. Sample Population

We conducted a retrospective analysis of serological data from 426.528 unique patients referred to Synevo laboratories for HBsAg screening tests between January 2018 and December 2023. The patients examined were between less than 1 month and 99 years old. To investigate the geographical distribution of the HBV infection and co-infection in the Romanian population, we included in our study four geographical regions from all over Romania: western, eastern, southern, and central (including Bucharest). Following the routing ordering process, all patients signed an informed consent form, and blood samples were collected by specialized nurses.

### 2.2. Laboratory Testing

For each patient, following blood collection, serum was separated, and specific serological markers of hepatitis B, C, delta virus, and HIV were performed in Synevo laboratories according to the manufacturer’s instructions provided by the commercial kits and internal specific operational procedures.

#### 2.2.1. HBsAg, HBeAg, Anti-HBc IgM and Total, Anti-HBe, Anti-HBs

Markers of hepatitis B virus infection, including HBsAg, HBeAg, anti-HBc IgM and total, anti-HBe, and anti-HBs, were analyzed using Elecsys kits based on the electrochemiluminescence immunoassay (ECLIA) for the Cobas e801 immunoassay analyzers (Roche Diagnostics GmbH, Mannheim, Germany). The reagents used included Elecsys HBsAg II, Elecsys HBsAg II Auto Confirm, Elecsys HBeAg, Elecsys Anti-HBe, Elecsys Anti-HBc II, Elecsys Anti-HBc IgM, and Elecsys Anti-HBs II, all supplied by Roche Diagnostics. Results were automatically determined by the software.

The serological markers of HBV detected using the Elecsys 2010 (Roche, Mannheim, Germany) have a lower limit of detection of ≤0.04 U/mL (according to the recommendations of the Paul-Ehrlich-Institute, Germany, PEI standards) or ≤0.1 IU/mL (WHO International Standards) for HBsAg. Following standard operational procedures at the Synevo laboratory, all patients with HBsAg values between 1 and 50 were retested using the confirmatory Elecsys HBsAg II Auto Confirm assay. This confirmatory test involves an additional incubation phase with two parallel measurements based on specific antibody neutralization. The confirmation result (%) was automatically calculated by determining the ratio of the cutoff index for these two measurements. Results confirmed through antibody neutralization were considered positive for HBsAg if the control reaction COI was ≥0.81 and the confirmation result was ≤60%, with a lower limit of detection of 0.045 IU/mL.

For anti-HBs antibodies, used in monitoring the course of acute hepatitis B infection but also to measure the antibody status after vaccination, positive results were considered based on a cutoff index of ≥10 IU/L, with a lower limit of detection of <2 IU/L.

In conjunction with HBsAg and anti-HBs antibodies, which helped us investigate the external envelope of the hepatitis B virus, we also used Elecsys kits for detecting antibodies against the inner core antigen (HBcAg) (Roche Diagnostics GmbH, Mannheim, Germany). Anti-HBc total and IgM antibodies were investigated to differentiate between existing or prior infections and to diagnose hepatitis B in the absence of other markers (HBsAg-negative persons). The lower limit of detection was ≤0.8 WHO IU/mL for anti-HBc total, and samples with a cutoff index of ≤1 were considered reactive, after being tested in duplicate. For the Elecsys anti-HBc IgM, the cutoff sensitivity was 100 PEI-U/mL (according to the recommendations of the Paul-Ehrlich-Institute, Germany), and reactive samples, positive for anti-HBc IgM, were defined based on a cutoff index of ≥1.

For measuring HBeAg seroconversion to anti-HBe as markers of active viral replication, we investigated HBeAg, with a lower limit of detection (cutoff sensitivity) of ≤0.3 IU/mL and a cutoff index for positive results of ≥1.0, and anti-HBe with a lower limit of detection of <0.2 IU/mL.

#### 2.2.2. Anti-HCV

To test for antibodies against the hepatitis C virus, we utilized a third-generation assay, the Elecsys anti-HCV II assay, which is an electrochemiluminescence immunoassay (ECLIA) based on the sandwich principle. This was conducted on automated Cobas e 801 immunochemistry analyzers (Roche Diagnostics GmbH, Mannheim, Germany). In accordance with our standard operational procedures at the Synevo laboratory, all patients with anti-HCV values between 1 and 30 underwent confirmation through additional methods, such as immunoblotting or detection of HCV viral RNA.

#### 2.2.3. Anti-HIV 1 and 2 (Combi—Ag and Antibodies)

Elecsys HIV Duo immunoassay was used to investigate HIV-1 p24 antigen (HIV Ag), as well as antibodies to HIV-1 and HIV-2 (anti-HIV). The assay is based on the sandwich principle and performed using electrochemiluminescence on the automated Cobas e 801 immunochemistry analyzer (Roche Diagnostics GmbH, Mannheim, Germany). Samples with COI ≥ 1.00 were considered reactive and required to be confirmed through Western blot and HIV RNA testing. The cutoff sensitivity for HIV-1 p24 antigen was ≤1.0 IU/mL based on WHO International Standards, with no internationally accepted standard for limits and ranges of HIV-specific antibody detection. All reactive samples were also tested by the ELFA method.

#### 2.2.4. Anti-HDV Antibody Testing

For the qualitative detection of anti-HDV antibodies (IgG and IgM), we utilized the LIAISON^®^ XL murex Anti-HDV assay based on chemiluminescence (CLIA) technology (DIASORIN Biotechnology, Sallugia, Italy), with a lower limit of detection of 0.100 AU/mL. Reactive samples for anti-HDV antibodies were defined based on a cutoff of ≥1 AU/mL.

### 2.3. Statistical Analysis

Statistical processing of data was performed using the r Pearson correlation coefficient.

## 3. Results

### 3.1. Study Group Characteristics

**Figure 1:**
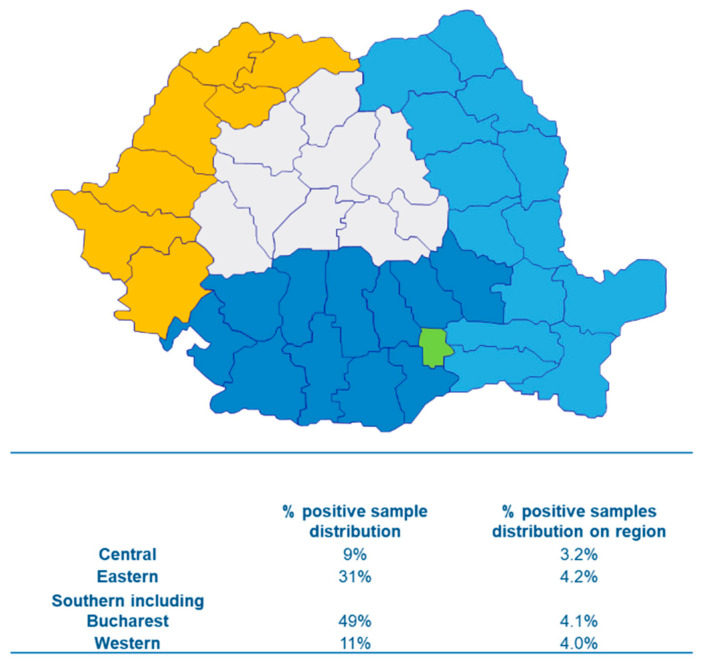
Sample cohort divided by Romanian region. Samples were collected from patients residing in various areas of Romania and categorized into distinct geographic regions: western (orange), central (white), eastern (light blue), southern (blue) and Bucharest (green). The distribution of samples across these regions is shown, with the southern region contributing the largest number of samples.

A total of 426,528 unique patients registered in the Synevo laboratory information system between January 2018 and December 2023 were included in this study after being referred for HBsAg testing. All persons were living in Romania, aged between less than 1 month to 99 years (Figure 1). The samples were referred by clinicians located all over Romania, which was broadly categorized into four geographical regions: western, eastern, southern, and central (including Bucharest) (Figure 1).

### 3.2. Characterization of Positive HBsAg Population

**Figure 2:**
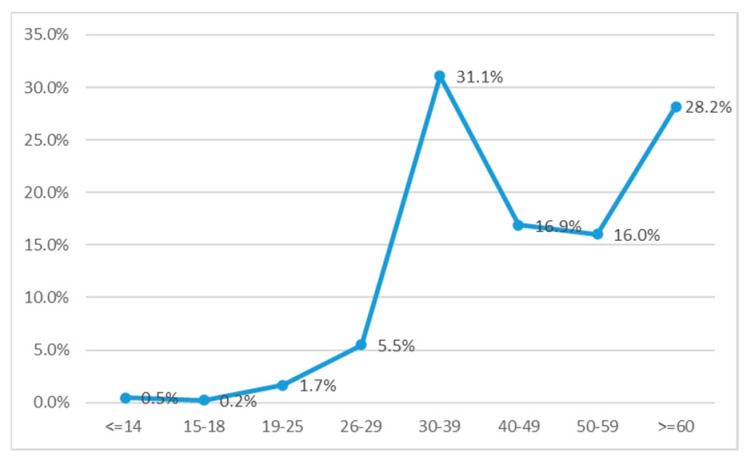
Age distribution of HBsAg-positive samples.

Out of the total of 426,528 unique patients, 17,082 were found positive for HBsAg with an overall HBV infection prevalence of 4.0%. The patients were categorized into eight subgroups based on age (<14, 15–18, 19–25, 26–29, 30–39, 40–49, 50–59, and >60 years). We observed the highest percentage of positivity in two subgroups 30–39 years and over 60 years, with a decrease in the groups aged 30–49 and 50–59 yrs (Figure 2).

Among participants who were in the groups with the highest positivity rate (30–39 and over 60 years), HBV prevalence in males was 16.4% and 14.5%, respectively, vs. 14.7% and 13.7%, respectively, in females.

**Figure 3:**
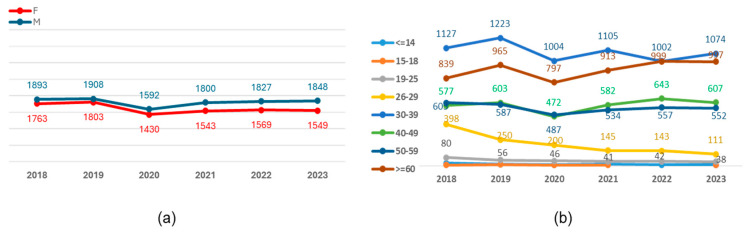
Distribution of the sample cohort positives for HBsAg on gender (**a**) and age subgroups (**b**), between 2018 and 2023.

We also examined the trends in infection rates from 2018 to 2023 across gender and age subgroups. Our analysis revealed a consistent number of cases, averaging 3444 per year, with a nearly equal distribution between men (53%) and women (47%) (Figure 3).

### 3.3. HVB Infection Staging

For the 17,082 positive samples sent to Synevo laboratories, we analyzed additional serological markers, including anti-HBc IgM and HBeAg for acute infection, as well as HBeAg and total anti-HBc or the absence of anti-HBc IgM for chronic infection. These tests were requested by clinicians either as part of initial co-testing or conducted within a two-month period.

Among the 17,082 patients, 646 were tested for anti-HBc IgM antibodies, with only 36 positive cases indicating acute infection, representing 0.2% of the total positive population. Additionally, 0.56% of the HBsAg-positive cohort was found to be positive for the HBeAg marker.

To characterize chronic infection, we analyzed total anti-HBc antibodies and HBeAg, identifying 2263 positive samples for anti-HBc antibodies, which accounts for 13.2% of the total positive cohort. Within the chronic infection group, we observed a stable patient population from 2018 to 2023, with the highest prevalence found in the 30–39 age subgroup (Table 1).

Since the detection of HBeAg and anti-HBe is crucial for determining the phases of chronic hepatitis B (CHB) infection and is linked to active HBV replication and an increased risk of hepatocellular carcinoma (HCC), we assessed their presence in the chronic infection population. Among the positive cohort, 15.4% were referred for HBeAg testing and 14% for anti-HBe antibodies. The positivity rates were 3.6% for HBeAg and 95.4% for anti-HBe antibodies (Table 2).

For 30% of the positive samples, the clinician also requested viral load, and 25.5% had a detectable HBV DNA. Among these patients, 55.7% were referred for liver enzyme testing, with 81.4% and 90.3% showing normal ALT and AST values, respectively. We ran a correlation analysis between ALT and AST values and viral load, and an r Pearson correlation coefficient of 0.07 indicated a very weak positive correlation (Table 3).

Twenty-five percent of patients tested for ALT serum level exhibited abnormal results. Of these, 21% had values ranging from one to three times the upper limit of the reference range (Table 4).

### 3.4. Co-Infection HBV and HDV

**Figure 4:**
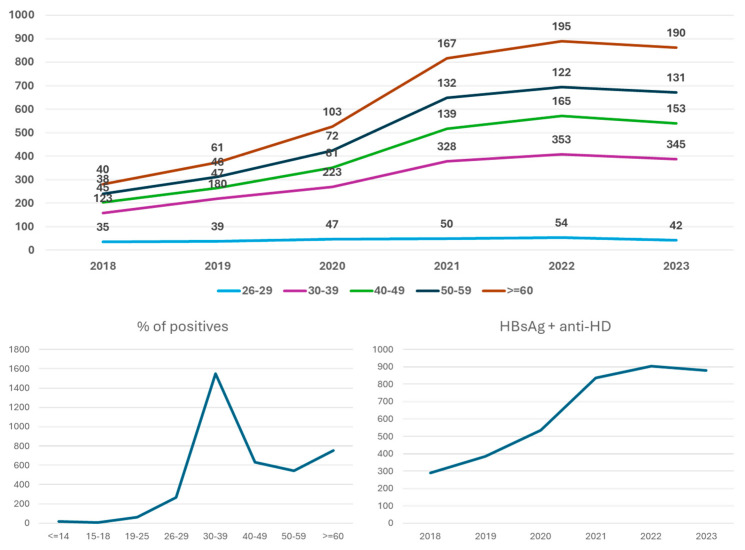
Distribution of the anti-HVD positive samples on age subgroups and the period from 2018 to 2023, when it was observed that the number of co-infection cases had tripled by 2023.

For 55% of the HBV-positive patients (3229 individuals), clinicians also requested anti-HD antibody testing. Out of the total 17,082 samples, 1922 tested positive, representing 11.3% of the cohort (Table 5). The gender distribution was 46% female and 54% male. During the period from 2018 to 2023, we observed a significant increase in cases with age, with the number of co-infection cases tripling by 2023 compared to 2018. The largest number of patients with co-infection was found in the 30–39 and over 60 age subgroups (Figure 4).

### 3.5. Co-Infection HBV and HCV

**Figure 5:**
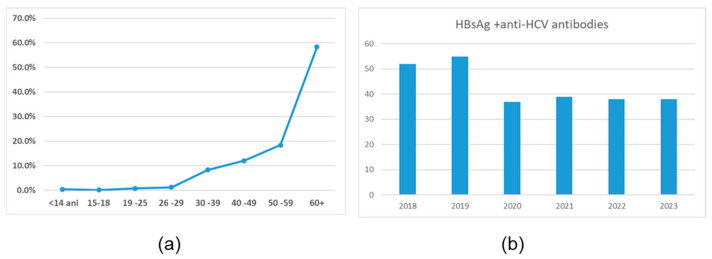
Characterization of the population with HBV and HCV co-infection. Positivity rate (1.4%) for anti-HCV in the HbsAg-positive population. Distribution of the anti-HCV positive samples on age subgroups (**a**) and between 2018 and 2023 (**b**).

In the population positive for HBsAg, those co-infected with HCV accounted for 1.4% (232 patients), with a nearly equal distribution between genders (53% female, 47% male) (Table 6). The age distribution was as follows: 8.4% in the 30–39 age group, 12.1% in 40–49, 18.5% in 50–59, and 58.4% in those over 60. The number of patients with both HBV and HCV co-infection has declined since 2020, stabilizing at 38 cases per year (38 ± 1) from 2020 to 2023, predominantly among individuals over 60. Additionally, 55 unique patients tested positive for HbsAg, and anti-HCV were referred for hepatitis B or C viral load testing. Among them, 14% had a detectable viral load for hepatitis B, while 6% had detectable hepatitis C (Figure 5).

### 3.6. Co-Infection HBV and HIV

**Figure 6:**
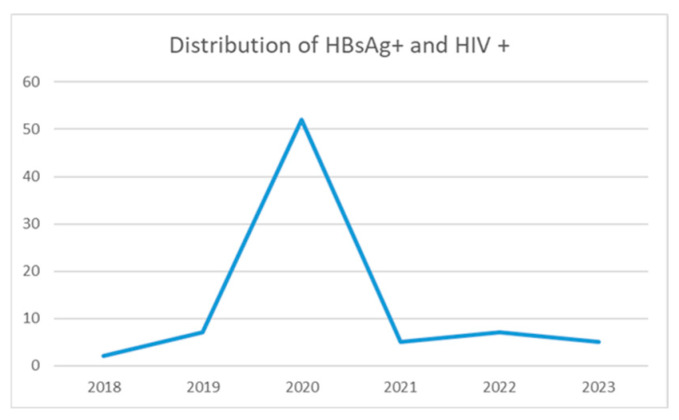
Distribution of the anti-HIV positive samples between 2018 and 2023.

We also analyzed the co-existence of HIV and HBV infections in the population positive for HbsAg and found that 0.45% were also HIV positive (Table 7). The evolution of the number of co-infection cases detected between 2018-2023 is shown in Figure 6.

## 4. Discussion

Although an effective preventive hepatitis B vaccine has been available for over 30 years [9], the amount of newly detected cases of hepatitis B infections in various European countries stays elevated, with most of them being categorized as chronic which is a major cause of chronic liver disease. Epidemiologic studies have shown that the prevalence of HDV/HBV co-infection varies globally, influenced by geographical, socio-economic, and demographic factors [9,10,11]. Considering these data, our study synthesizes findings from a study of 426,528 patients referred for HBsAg testing in Romania between 2018 and 2023, highlighting regional, gender, and age distributions of HBV infections, the evolution of infection rates, and co-infections with hepatitis C (HCV), hepatitis D (HDV), and HIV.

In our cohort, the overall prevalence of HBsAg positivity was 4.0%, with 17,082 patients testing positive for HBV. This prevalence aligns with previous reports indicating that Eastern Europe has a significant burden of HBV infection [10,11,12,13]. A study conducted by ECDC estimated HBV prevalence in Europe to vary between 0.5% and 6%, with Romania still showing a high prevalence rate [3]. In Romania, the historical prevalence of HBV, combined with socio-economic factors, plays a role in the regional differences observed, especially in underserved areas [14,15].

Interestingly, our data suggest that the highest rates of infection occurred in individuals aged 30–39 and those over 60. This bimodal distribution is consistent with global trends observed in endemic regions, where older populations are more likely to have been exposed to the virus before the implementation of universal vaccination programs, while middle-aged adults may have acquired infections due to suboptimal vaccination coverage or exposure in adulthood [16,17]. The relatively lower prevalence in the 40–49 and 50–59 age groups may reflect the success of earlier intervention strategies aimed at curbing HBV transmission in these populations [18].

Over the last five years, our study revealed a consistent number of HBV cases, averaging around 3444 annually. The gender distribution of HBV positivity, with a slight male predominance, is consistent with global trends, where males typically exhibit higher rates of HBV infection compared to females [16,17,18]. This male predominance may be attributed to behavioral factors, including higher rates of risky behaviors associated with viral transmission, as well as biological factors that may influence susceptibility to infection [19,20,21,22].

The absence of an increase in cases could suggest effective screening and management practices; however, it may also point to ongoing risks in the community that have yet to be addressed.

Comparative studies highlight that while some SEE countries have taken steps to reduce HBV prevalence through improved vaccination coverage and access to antiviral therapies, others have stagnated [23]. Romania’s approach, with consistent vaccination programs and screening practices over the years, exhibits the importance of regular surveillance in managing HBV.

The study’s analysis of serological markers for HBV infection staging revealed that a small percentage of patients presented with acute infections, while the majority were chronic cases. The low percentage of acute infections (0.2%) suggests that the cohort is predominantly composed of individuals with chronic HBV, which is a common scenario in HBV epidemiology [19,24].

Chronic HBV infection, characterized by the presence of total anti-HBc antibodies was identified in 13.2% of the positive population. This figure is significant as chronic HBV infection carries a higher risk of developing complications such as cirrhosis and hepatocellular carcinoma (HCC) [25]. The stable number of chronic HBV cases between 2018 and 2023 suggests a persistent burden of chronic HBV in Romania, particularly among the 30–39 age group, which demonstrated the highest prevalence. This finding is consistent with global data indicating that individuals infected at younger ages, especially perinatally or during early childhood, are more likely to develop chronic HBV infection.

HBeAg positivity, a marker of active viral replication and an increased risk for progression to liver disease, was observed in 0.6% of the chronic infection cohort. Conversely, anti-HBe antibodies, which signify a lower replicative state and better prognosis, were detected in 95.4% of those tested. This distribution indicates that while a small proportion of chronic HBV patients have active viral replication, the majority are in the immune control phase, which is associated with lower HBV DNA levels and a reduced risk of complications. The correlation between liver enzyme levels and viral load, although weak, highlights the complexity of HBV infection dynamics and the necessity for comprehensive monitoring of liver function in affected individuals [19,25].

Recently, an increase in HDV transmission has been noted in certain Western European countries, particularly among intravenous drug users and immigrants from Eastern Europe and Sub-Saharan Africa. Globally, approximately 5% of patients with hepatitis B also develop hepatitis D, leading to more severe outcomes due to the rapid progression of liver disease [26,27,28]. Compared to those with HBV infection alone, patients co-infected with HDV and HBV are three times more likely to progress from cirrhosis to hepatocellular carcinoma [26,29].

The increase in HDV co-infection rates over the study period, with a threefold rise from 2018 to 2023, which aligns with the literature [29], is alarming and suggests that this co-infection remains under-recognized and under-managed. The implications of co-infections are significant, as they are associated with higher morbidity and mortality rates, particularly due to the compounded effects on liver disease progression and the increased risk of hepatocellular carcinoma (HCC) [27]. This trend emphasizes the importance of screening for HDV in HBV-infected patients, as the presence of HDV can lead to more severe liver disease outcomes [29,30,31]. The increasing prevalence of HDV co-infection in Romania reflects similar trends observed in other regions, where HDV poses a significant public health challenge due to its association with more aggressive forms of hepatitis [14].

The age-related increase in co-infection rates underlines the necessity of integrating HCV and HDV robust screening in routine HBV management protocols.

The prevalence of HCV co-infection among HBV-positive patients was found to be 1.4%, with a notable age-related distribution skewed toward older adults. Interestingly, the study notes a steady decline in HBV/HCV co-infection cases from 2020 onward, with an average of 38 cases per year between 2020 and 2023. This figure is consistent with declining HCV prevalence in SEE countries due to improved screening, public health initiatives, and the introduction of highly effective therapies for HCV, which reduce viral load and transmission risk [32,33]. The highest proportion of HBV/HCV co-infected patients was found in the over 60 years age group (58.4%), reflecting the historical burden of HCV among older adults who may have been exposed before blood safety measures and harm reduction programs were in place [13,34].

The relatively low viral load detection in co-infected patients (14% for HBV and 6% for HCV) suggests that these individuals may be benefiting from effective antiviral therapies, particularly for HCV, which transformed the treatment landscape.

In our analysis, the co-infection rate of HBV and HIV was found to be 0.45%. This rate is lower than reported figures in other parts of Europe, where co-infection rates among patients with HIV are approximately 7.4% [35,36]. The lower prevalence in Romania may reflect differences in the population demographics, access to HIV care, and the impact of targeted interventions in reducing HIV transmission rates [35]. It is critical to note that individuals co-infected with HBV and HIV face unique challenges in treatment and disease management. The interaction between these viruses requires a multidisciplinary approach to treatment, combining antiviral therapy with regular monitoring to assess liver disease progression [37,38].

Additionally, the increasing prevalence of co-infections needs a multidisciplinary treatment approach that includes regular monitoring of liver function and viral loads. This approach should be complemented by educational initiatives aimed at reducing stigma and promoting safer practices among at-risk populations [39,40].

Despite the extensive nature of the study, it has some limitations, particularly the lack of clinical data (signs, symptoms, personal and family history). Information regarding the reasons for patient referral, such as routine screening or due to symptoms or clinical suspicion, was not available. Additionally, the study did not capture data regarding the modes of transmission, such as drug abuse or sexual transmission, which are crucial factors for understanding the epidemiology of co-infections and for tailoring public health interventions.

## 5. Conclusions

The epidemiological patterns of HBV infection and its co-infections with HDV, HCV, and HIV in Romania, as observed in this study, reflect both successes and ongoing challenges in the management of viral hepatitis.

The overall HBV prevalence of 4% is indicative of a high endemic burden, and particular attention is needed for ongoing surveillance, targeted interventions, and integrated care strategies to effectively manage hepatitis B and its co-infections, especially for chronic HBV carriers who remain at risk of developing severe liver disease.

Enhanced awareness and proactive management of co-infections, particularly with HDV and HCV, are crucial for improving patient outcomes and reducing the overall impact of viral hepatitis on public health. Research that will include detailed clinical data, the reasons for referral, and modes of transmission will provide a more comprehensive understanding of the epidemiology of HBV and its co-infections, as well as guide more targeted public health strategies.

Future studies should also aim to explore the psychosocial factors influencing risk behaviors, vaccination uptake, and treatment adherence, thereby enriching our understanding of the multifaceted challenges posed by HBV and its co-infections in SEE Europe.

## Figures and Tables

**Table 1:** Age subgroup distribution of HbsAg and anti-HBs-positive samples (**a**). A total of 13.2% of the total HBsAg-positive cohort had chronic infection (**b**). Distribution of chronic infection during 2018–2023 (**c**).

(a)							
HBsAg+	Anti–HBc+	≤14		14		1%	
		15–18		6		0.3%	
		19–25		37		2%	
		26–29		135		6%	
		30–39		878		39%	
		40–49		348		15%	
		50–59		321		14%	
		≥60		524		23%	
**(b)**							
		2018	2019	2020	2021	2022	2023
HBsAg+ (17,082)		3662	3713	3131	3349	3405	3402
anti-HBc+ (2263)		376	359	367	418	397	443
		10.3%	9.7%	11.72%	12.5%	11.7%	13.0%
**(c)**							
HBsAg+	Anti–HBc+	F		1069		47%	
		M		1193		53%	

**Table 2:** Distribution of HBeAg (**a**) and anti-Hbe (**b**) antibodies among HbsAg-positive samples. The rates of positivity were 3.6% for HBeAg and 95.4% for anti-HBe antibodies.

(a)		
HBsAg+	HBeAg+	anti-HBc+
17,082	97	36
	HBeAg+	anti-HBc+
	2536	1071
**(b)**		
HBsAg+	anti-HBe+	anti-HBc+
17,082	2299	1015
	anti-HBe−	anti-HBc+
	103	33

**Table 3:** The analysis of ALT, AST, De Ritis ratio (AST/ALT), and DNA VHB in HbsAg-positive patients with chronic and acute infection.

	HbsAg-NEGATIVE	HbsAg+	HbsAg+
		(HBsAg+ Anti-HBc IgM+)	(HBsAg+ Anti-HBc+)
**Total** **number of patients**	409,445	36	2263
**ALT**			
Unique patients	222,870	15	1218
Mean	31.3	650.3	53.4
Median	19.0	262.2	26.0
Standard deviation	92.9	919.9	230.1
**AST**			
Unique patients	219,306	16	1211
Mean	28.3	476.6	47.2
Median	20.0	148.0	24.0
Standard deviation	100.4	589.5	210.9
**De Ritis ratio (AST/ALT)**			
Unique patients	215,825	15	1194
Mean	1.12	0.92	1.00
Median	1.04	0.78	0.91
Standard deviation	0.65	0.56	0.48
Min-Max	0.08–118.2	0.3–2.9	0.14–10.39
**DNA VHB**			
Unique patients	8	5	548
Mean	25	84,086,975	9,461,893
Median	20	1480	666
Standard deviation	22	222,204,871	98,278,438
Min-Max	10–78	10–5.88 × 10^8^	10–1.84 × 10^8^

**Table 4:** ALT and AST variations among HBsAg-positive and -negative patients.

		Number of Patients HBsAg Negative	Number of Patients HBsAg Positive
GPT/ALAT/ALT	1–3 × N	32,727	635
	3–5 × N	2826	45
	5–10 × N	1421	21
	≥10 × N	1031	25
	N	189,903	2190
GOT/ASAT/AST	1–3 × N	17,688	330
	3–5 × N	1852	33
	5–10 × N	1041	16
	≥10 × N	738	16
	N	200,891	2400

**Table 5:** Characterization of the population with HBV and HDV co-infection. Positivity rate (11.3%) for anti-HVD in HbsAg-positive population.

Anti-HD		3229
	Negative	1568
	Positive	1922

**Table 6:** Characterization of the population with HBV and HCV co-infection. Positivity rate (1.4%) for anti-HCV in the HbsAg-positive population.

HbsAg +	Anti-HCV	
	Negative	10,737
	Positive	232

**Table 7:** Characterization of the population with HBV and HIV co-infection. Positivity rate (1.4%) for anti-HIV in the HbsAg-positive population. Distribution of the anti-HIV positive samples on age subgroups.

Age Groups (Years)	% of HIV Positive
19–25	21
26–29	13
30–39	35
40–49	13
50–59	5
≥60	5

## Data Availability

The original contributions presented in the study are included in the article, further inquiries can be directed to the corresponding author.
